# From correlation to causation networks: a simple approximate learning algorithm and its application to high-dimensional plant gene expression data

**DOI:** 10.1186/1752-0509-1-37

**Published:** 2007-08-06

**Authors:** Rainer Opgen-Rhein, Korbinian Strimmer

**Affiliations:** 1 Department of Statistics, Ludwig-Maximilians-Universität München, Ludwigstraße 33, D-80539 München, Germany; 2 Institute for Medical Informatics, Statistics and Epidemiology (IMISE), University of Leipzig, Härtelstr. 16-18, 04107 Leipzig, Germany

## Abstract

**Background:**

The use of correlation networks is widespread in the analysis of gene expression and proteomics data, even though it is known that correlations not only confound direct and indirect associations but also provide no means to distinguish between cause and effect. For "causal" analysis typically the inference of a directed graphical model is required. However, this is rather difficult due to the curse of dimensionality.

**Results:**

We propose a simple heuristic for the statistical learning of a high-dimensional "causal" network. The method first converts a correlation network into a partial correlation graph. Subsequently, a partial ordering of the nodes is established by multiple testing of the log-ratio of standardized partial variances. This allows identifying a directed acyclic causal network as a subgraph of the partial correlation network. We illustrate the approach by analyzing a large *Arabidopsis thaliana *expression data set.

**Conclusion:**

The proposed approach is a heuristic algorithm that is based on a number of approximations, such as substituting lower order partial correlations by full order partial correlations. Nevertheless, for small samples and for sparse networks the algorithm not only yield sensible first order approximations of the causal structure in high-dimensional genomic data but is also computationally highly efficient.

**Availability and Requirements:**

The method is implemented in the "GeneNet" R package (version 1.2.0), available from CRAN and from . The software includes an R script for reproducing the network analysis of the *Arabidopsis thaliana *data.

## Background

Correlation networks are widely used to explore and visualize high-dimensional data, for instance in finance [1-3], ecology [4], gene expression analysis [5,6], or metabolomics [7]. Their popularity is owed to a large extent to the ease with which a correlation network can be constructed, as this requires only two simple steps: i) the computation of all pairwise correlations for the investigated variables, and ii) a thresholding or filtering procedure [8] to identify significant correlations, and hence edges, of the network.

However, for shedding light on the causal processes underlying the observed data, correlation networks are only of limited use. This is due to the fact that correlations not only confound direct and indirect associations but also provide no means to distinguish between response variables and covariates (and thus between cause and effect).

Therefore, causal analysis requires tools different from correlation networks: much of the work in this area has focused on Bayesian networks [9] or related regression models such as systems of recursive equations [10,11] or influence diagrams [12]. All of these models have in common that they describe causal relations by an underlying directed acyclic graph (DAG).

There already exist numerous methods for learning DAGs from observational data – see for instance the summarizing review in [13] and the references therein. However, with few exceptions [e.g., the PC algorithm, [14,15]] virtually all of these methods have been devised for comparatively small numbers of variables and with large sample size in mind. For instance, the numerical example of the recently proposed algorithm described in [16] uses *n *= 10,000 observations for *p *= 7 variables. Unfortunately, the data that would be most interesting to explore with causal methods, namely those commonly visualized by correlation networks (see above), have completely different characteristics, in particular they are likely of high dimension.

In this paper we follow [15] and focus on modeling large-scale linear recursive systems. Specifically, we present a simple discovery algorithm that enables the inference of causal relations from small sampled data and for large numbers of variables. It proceeds in two steps as follows:

• First, the correlation network is transformed into a partial correlation network, which is essentially an undirected graph that displays the direct linear associations only. This type of network model is also known under the names of graphical Gaussian model (GGM), concentration graph, covariance selection graph, conditional independence graph (CIG), or Markov random field. Note that there is a simple relationship between correlation and partial correlation. Moreover, in recent years there has been much progress with regard to statistical methodology for learning large-scale partial correlation graphs from small samples [e.g., [17-22]]. Here we employ the approach described in [20].

• Second, the undirected GGM is converted into a *partially *directed graph. This is done by estimating a pairwise ordering of the nodes from the data using multiple testing of the log-ratios of standardized partial variances, and by subsequent projection of this partial ordering onto the GGM. The inferred causal network is the subgraph containing all the directed edges.

Note that this algorithm is similar to the PC algorithm in that edges are being removed from the independence graph to obtain the underlying DAG. However, our criterion for eliminating an edge is distinctly different from that of the PC algorithm.

The remainder of the paper is organized as follows. First, we describe the methodology. Second we consider its statistical interpretation and further properties. Subsequently, we illustrate the approach by analyzing an 800 gene data set from a large-scale *Arabidopsis thaliana *gene expression experiment. Finally, we conclude with some discussion of the method, commenting also on the limitations of the approach.

## Methods

### Theoretical basis

Consider a linear regression with *Y *as response and *X*_1_, ..., *X*_*k*_, ..., *X*_*K *_as covariates. We assume that *X*_*k *_and *Y *are random variables with known variances var(*Y*) and var(*X*_*k*_) and with covariance cov(*Y*, *X*_*k*_). The best linear predictor of *Y *in terms of the *X*_*k *_that minimizes the MSE of ∑_*k *_*β*_*k*_*X*_*k *_- *Y *is given by [e.g. ref. [23], p. 206]

βky=ρ˜ykσ˜y2σ˜k2,
 MathType@MTEF@5@5@+=feaafiart1ev1aaatCvAUfKttLearuWrP9MDH5MBPbIqV92AaeXatLxBI9gBaebbnrfifHhDYfgasaacH8akY=wiFfYdH8Gipec8Eeeu0xXdbba9frFj0=OqFfea0dXdd9vqai=hGuQ8kuc9pgc9s8qqaq=dirpe0xb9q8qiLsFr0=vr0=vr0dc8meaabaqaciaacaGaaeqabaqabeGadaaakeaaiiGacqWFYoGydaqhaaWcbaGaem4AaSgabaGaemyEaKhaaOGaeyypa0Jaf8xWdiNbaGaadaWgaaWcbaGaemyEaKNaem4AaSgabeaakmaakaaabaWaaSaaaeaacuWFdpWCgaacamaaDaaaleaacqWG5bqEaeaacqaIYaGmaaaakeaacuWFdpWCgaacamaaDaaaleaacqWGRbWAaeaacqaIYaGmaaaaaaqabaGccqGGSaalaaa@410B@

where ρ˜yk
 MathType@MTEF@5@5@+=feaafiart1ev1aaatCvAUfKttLearuWrP9MDH5MBPbIqV92AaeXatLxBI9gBaebbnrfifHhDYfgasaacH8akY=wiFfYdH8Gipec8Eeeu0xXdbba9frFj0=OqFfea0dXdd9vqai=hGuQ8kuc9pgc9s8qqaq=dirpe0xb9q8qiLsFr0=vr0=vr0dc8meaabaqaciaacaGaaeqabaqabeGadaaakeaaiiGacuWFbpGCgaacamaaBaaaleaacqWG5bqEcqWGRbWAaeqaaaaa@3188@ and is the *partial *correlation between *Y *and *X*_*k*_, and σ˜y2
 MathType@MTEF@5@5@+=feaafiart1ev1aaatCvAUfKttLearuWrP9MDH5MBPbIqV92AaeXatLxBI9gBaebbnrfifHhDYfgasaacH8akY=wiFfYdH8Gipec8Eeeu0xXdbba9frFj0=OqFfea0dXdd9vqai=hGuQ8kuc9pgc9s8qqaq=dirpe0xb9q8qiLsFr0=vr0=vr0dc8meaabaqaciaacaGaaeqabaqabeGadaaakeaaiiGacuWFdpWCgaacamaaDaaaleaacqWG5bqEaeaacqaIYaGmaaaaaa@311F@ and σ˜k2
 MathType@MTEF@5@5@+=feaafiart1ev1aaatCvAUfKttLearuWrP9MDH5MBPbIqV92AaeXatLxBI9gBaebbnrfifHhDYfgasaacH8akY=wiFfYdH8Gipec8Eeeu0xXdbba9frFj0=OqFfea0dXdd9vqai=hGuQ8kuc9pgc9s8qqaq=dirpe0xb9q8qiLsFr0=vr0=vr0dc8meaabaqaciaacaGaaeqabaqabeGadaaakeaaiiGacuWFdpWCgaacamaaDaaaleaacqWGRbWAaeaacqaIYaGmaaaaaa@3103@ are the respective *partial *variances. 

The partial correlation is the correlation that remains between two variables if the effect of the other variables has been regressed away. Likewise, the partial variance is the variance that remains if the influences of all other variables are taken into account. Table 1 lists the definitions and formulas for the computation of these quantities (note that in our notation a tilde on top of a symbol indicates ''partial'').

**Table 1:** Formulas for computing partial variances and partial correlations

	Definition	True value	Estimate
Covariance matrix:	cov(*X*_*k*_, *X*_*l*_) = *σ*_*kl*_	**Σ **= (*σ*_*kl*_)	***S ***= (*s*_*kl*_)
Concentration matrix:	**Ω **= **Σ**^-1^	**Ω **= (*ω*_*kl*_)	
Variances:	var(*X*_*k*_) = *σ*_*kk *_= σk2 MathType@MTEF@5@5@+=feaafiart1ev1aaatCvAUfKttLearuWrP9MDH5MBPbIqV92AaeXatLxBI9gBaebbnrfifHhDYfgasaacH8akY=wiFfYdH8Gipec8Eeeu0xXdbba9frFj0=OqFfea0dXdd9vqai=hGuQ8kuc9pgc9s8qqaq=dirpe0xb9q8qiLsFr0=vr0=vr0dc8meaabaqaciaacaGaaeqabaqabeGadaaakeaaiiGacqWFdpWCdaqhaaWcbaGaem4AaSgabaGaeGOmaidaaaaa@30F4@	*σ*_*kk*_	*s*_*kk*_
Partial variances	var(*X*_*k*_|*X*_≠*k*_) = σ˜kk MathType@MTEF@5@5@+=feaafiart1ev1aaatCvAUfKttLearuWrP9MDH5MBPbIqV92AaeXatLxBI9gBaebbnrfifHhDYfgasaacH8akY=wiFfYdH8Gipec8Eeeu0xXdbba9frFj0=OqFfea0dXdd9vqai=hGuQ8kuc9pgc9s8qqaq=dirpe0xb9q8qiLsFr0=vr0=vr0dc8meaabaqaciaacaGaaeqabaqabeGadaaakeaaiiGacuWFdpWCgaacamaaBaaaleaacqWGRbWAcqWGRbWAaeqaaaaa@316F@ = σ˜k2 MathType@MTEF@5@5@+=feaafiart1ev1aaatCvAUfKttLearuWrP9MDH5MBPbIqV92AaeXatLxBI9gBaebbnrfifHhDYfgasaacH8akY=wiFfYdH8Gipec8Eeeu0xXdbba9frFj0=OqFfea0dXdd9vqai=hGuQ8kuc9pgc9s8qqaq=dirpe0xb9q8qiLsFr0=vr0=vr0dc8meaabaqaciaacaGaaeqabaqabeGadaaakeaaiiGacuWFdpWCgaacamaaDaaaleaacqWGRbWAaeaacqaIYaGmaaaaaa@3103@ = ωkk−1 MathType@MTEF@5@5@+=feaafiart1ev1aaatCvAUfKttLearuWrP9MDH5MBPbIqV92AaeXatLxBI9gBaebbnrfifHhDYfgasaacH8akY=wiFfYdH8Gipec8Eeeu0xXdbba9frFj0=OqFfea0dXdd9vqai=hGuQ8kuc9pgc9s8qqaq=dirpe0xb9q8qiLsFr0=vr0=vr0dc8meaabaqaciaacaGaaeqabaqabeGadaaakeaaiiGacqWFjpWDdaqhaaWcbaGaem4AaSMaem4AaSgabaGaeyOeI0IaeGymaedaaaaa@3348@	σ˜kk MathType@MTEF@5@5@+=feaafiart1ev1aaatCvAUfKttLearuWrP9MDH5MBPbIqV92AaeXatLxBI9gBaebbnrfifHhDYfgasaacH8akY=wiFfYdH8Gipec8Eeeu0xXdbba9frFj0=OqFfea0dXdd9vqai=hGuQ8kuc9pgc9s8qqaq=dirpe0xb9q8qiLsFr0=vr0=vr0dc8meaabaqaciaacaGaaeqabaqabeGadaaakeaaiiGacuWFdpWCgaacamaaBaaaleaacqWGRbWAcqWGRbWAaeqaaaaa@316F@	s˜kk MathType@MTEF@5@5@+=feaafiart1ev1aaatCvAUfKttLearuWrP9MDH5MBPbIqV92AaeXatLxBI9gBaebbnrfifHhDYfgasaacH8akY=wiFfYdH8Gipec8Eeeu0xXdbba9frFj0=OqFfea0dXdd9vqai=hGuQ8kuc9pgc9s8qqaq=dirpe0xb9q8qiLsFr0=vr0=vr0dc8meaabaqaciaacaGaaeqabaqabeGadaaakeaacuWGZbWCgaacamaaBaaaleaacqWGRbWAcqWGRbWAaeqaaaaa@3114@
Correlations:	corr(*X*_*k*_, *X*_*l*_) = *ρ*_*kl *_= *σ*_*kl *_(*σ*_*kk *_*σ*_*ll*_)^-1/2^	***P ***= (*ρ*_*kl*_)	***R ***= (*r*_*kl*_)
Partial correlations:	corr(*X*_*k*_, *X*_*l*_|*X*_≠*k*, *l*_) = ρ˜kl=−ωkl(ωkkωll)−1/2 MathType@MTEF@5@5@+=feaafiart1ev1aaatCvAUfKttLearuWrP9MDH5MBPbIqV92AaeXatLxBI9gBaebbnrfifHhDYfgasaacH8akY=wiFfYdH8Gipec8Eeeu0xXdbba9frFj0=OqFfea0dXdd9vqai=hGuQ8kuc9pgc9s8qqaq=dirpe0xb9q8qiLsFr0=vr0=vr0dc8meaabaqaciaacaGaaeqabaqabeGadaaakeaaiiGacuWFbpGCgaacamaaBaaaleaacqWGRbWAcqWGSbaBaeqaaOGaeyypa0JaeyOeI0Iae8xYdC3aaSbaaSqaaiabdUgaRjabdYgaSbqabaGccqGGOaakcqWFjpWDdaWgaaWcbaGaem4AaSMaem4AaSgabeaakiab=L8a3naaBaaaleaacqWGSbaBcqWGSbaBaeqaaOGaeiykaKYaaWbaaSqabeaacqGHsislcqaIXaqmcqGGVaWlcqaIYaGmaaaaaa@4739@	P˜=(ρ˜kl) MathType@MTEF@5@5@+=feaafiart1ev1aaatCvAUfKttLearuWrP9MDH5MBPbIqV92AaeXatLxBI9gBaebbnrfifHhDYfgasaacH8akY=wiFfYdH8Gipec8Eeeu0xXdbba9frFj0=OqFfea0dXdd9vqai=hGuQ8kuc9pgc9s8qqaq=dirpe0xb9q8qiLsFr0=vr0=vr0dc8meaabaqaciaacaGaaeqabaqabeGadaaakeaaieWacuWFqbaugaacaiabg2da9maabmaabaacciGaf4xWdiNbaGaadaWgaaWcbaGaem4AaSMaemiBaWgabeaaaOGaayjkaiaawMcaaaaa@3546@	R˜=(r˜kl) MathType@MTEF@5@5@+=feaafiart1ev1aaatCvAUfKttLearuWrP9MDH5MBPbIqV92AaeXatLxBI9gBaebbnrfifHhDYfgasaacH8akY=wiFfYdH8Gipec8Eeeu0xXdbba9frFj0=OqFfea0dXdd9vqai=hGuQ8kuc9pgc9s8qqaq=dirpe0xb9q8qiLsFr0=vr0=vr0dc8meaabaqaciaacaGaaeqabaqabeGadaaakeaaieWacuWFsbGugaacaiabg2da9maabmaabaGafmOCaiNbaGaadaWgaaWcbaGaem4AaSMaemiBaWgabeaaaOGaayjkaiaawMcaaaaa@34F1@

Index *i *runs from 1 to *n *(sample size), and indices *k *and *l *run from 1 to *p *(dimension). A tilde denotes a "partial" quantity.

From Equation 1 it is immediately clear that the complete linear system and thus all βky
 MathType@MTEF@5@5@+=feaafiart1ev1aaatCvAUfKttLearuWrP9MDH5MBPbIqV92AaeXatLxBI9gBaebbnrfifHhDYfgasaacH8akY=wiFfYdH8Gipec8Eeeu0xXdbba9frFj0=OqFfea0dXdd9vqai=hGuQ8kuc9pgc9s8qqaq=dirpe0xb9q8qiLsFr0=vr0=vr0dc8meaabaqaciaacaGaaeqabaqabeGadaaakeaaiiGacqWFYoGydaqhaaWcbaGaem4AaSgabaGaemyEaKhaaaaa@315B@ are determined by the joint covariance matrix of *Y *and *X*_*k *_[see also, e.g., [24,24]]. For only a single dependent variable Equation 1 reduces to the well-known relation βxy=ρyxσy2/σx2
 MathType@MTEF@5@5@+=feaafiart1ev1aaatCvAUfKttLearuWrP9MDH5MBPbIqV92AaeXatLxBI9gBaebbnrfifHhDYfgasaacH8akY=wiFfYdH8Gipec8Eeeu0xXdbba9frFj0=OqFfea0dXdd9vqai=hGuQ8kuc9pgc9s8qqaq=dirpe0xb9q8qiLsFr0=vr0=vr0dc8meaabaqaciaacaGaaeqabaqabeGadaaakeaaiiGacqWFYoGydaqhaaWcbaGaemiEaGhabaGaemyEaKhaaOGaeyypa0Jae8xWdi3aaSbaaSqaaiabdMha5jabdIha4bqabaGcdaGcaaqaaiab=n8aZnaaDaaaleaacqWG5bqEaeaacqaIYaGmaaGccqGGVaWlcqWFdpWCdaqhaaWcbaGaemiEaGhabaGaeGOmaidaaaqabaaaaa@4118@, which contains only the unconditioned correlation and variances (without the tilde).

We emphasize that Equation 1 has a direct relation with the usual ordinary least squares (OLS) estimator for the regression coefficient. This is recovered if the empirical covariance matrix is plugged into Equation 1. However, note that Equation 1 also remains valid if other estimates of the covariance are used, such as penalized or shrinkage estimators (note that there is no hat on βky
 MathType@MTEF@5@5@+=feaafiart1ev1aaatCvAUfKttLearuWrP9MDH5MBPbIqV92AaeXatLxBI9gBaebbnrfifHhDYfgasaacH8akY=wiFfYdH8Gipec8Eeeu0xXdbba9frFj0=OqFfea0dXdd9vqai=hGuQ8kuc9pgc9s8qqaq=dirpe0xb9q8qiLsFr0=vr0=vr0dc8meaabaqaciaacaGaaeqabaqabeGadaaakeaaiiGacqWFYoGydaqhaaWcbaGaem4AaSgabaGaemyEaKhaaaaa@315B@).

For the following it is important that Equation 1 can be further rewritten by introducing a scale factor. Specifically, by abbreviating the standardized partial variance σ˜k2/σk2
 MathType@MTEF@5@5@+=feaafiart1ev1aaatCvAUfKttLearuWrP9MDH5MBPbIqV92AaeXatLxBI9gBaebbnrfifHhDYfgasaacH8akY=wiFfYdH8Gipec8Eeeu0xXdbba9frFj0=OqFfea0dXdd9vqai=hGuQ8kuc9pgc9s8qqaq=dirpe0xb9q8qiLsFr0=vr0=vr0dc8meaabaqaciaacaGaaeqabaqabeGadaaakeaaiiGacuWFdpWCgaacamaaDaaaleaacqWGRbWAaeaacqaIYaGmaaGccqGGVaWlcqWFdpWCdaqhaaWcbaGaem4AaSgabaGaeGOmaidaaaaa@362F@ by SPV_*k*_, we can decompose the regression coefficient into the simple product

βky=ρ˜yk︸ASPVySPVk︸ℬσy2σk2︸C.
 MathType@MTEF@5@5@+=feaafiart1ev1aaatCvAUfKttLearuWrP9MDH5MBPbIqV92AaeXatLxBI9gBaebbnrfifHhDYfgasaacH8akY=wiFfYdH8Gipec8Eeeu0xXdbba9frFj0=OqFfea0dXdd9vqai=hGuQ8kuc9pgc9s8qqaq=dirpe0xb9q8qiLsFr0=vr0=vr0dc8meaabaqaciaacaGaaeqabaqabeGadaaakeaaiiGacqWFYoGydaqhaaWcbaGaem4AaSgabaGaemyEaKhaaOGaeyypa0ZaaGbaaeaacuWFbpGCgaacamaaBaaaleaacqWG5bqEcqWGRbWAaeqaaaqaamrtHrhAL1wy0L2yHvtyaeHbnfgDOvwBHrxAJfwnaGabaiab+bq8bbGccaGL44padaagaaqaamaakaaabaWaaSaaaeaacqqGtbWucqqGqbaucqqGwbGvdaWgaaWcbaGaemyEaKhabeaaaOqaaiabbofatjabbcfaqjabbAfawnaaBaaaleaacqWGRbWAaeqaaaaaaeqaaaqaaiab+XsicbGccaGL44padaagaaqaamaakaaabaWaaSaaaeaacqWFdpWCdaqhaaWcbaGaemyEaKhabaGaeGOmaidaaaGcbaGae83Wdm3aa0baaSqaaiabdUgaRbqaaiabikdaYaaaaaaabeaaaeaacqGFce=qaOGaayjo+dGaeiOla4caaa@5F66@

Note that SPV_*y *_and SPV_*k *_take on values from 0 to 1. All three factors have an immediate and intuitive interpretation:

A
 MathType@MTEF@5@5@+=feaafiart1ev1aaatCvAUfKttLearuWrP9MDH5MBPbIqV92AaeXatLxBI9gBaebbnrfifHhDYfgasaacH8akY=wiFfYdH8Gipec8Eeeu0xXdbba9frFj0=OqFfea0dXdd9vqai=hGuQ8kuc9pgc9s8qqaq=dirpe0xb9q8qiLsFr0=vr0=vr0dc8meaabaqaciaacaGaaeqabaqabeGadaaakeaat0uy0HwzTfgDPnwy1egaryqtHrhAL1wy0L2yHvdaiqaaliab=bq8bbaa@382B@ : This factor determines whether there is a direct association between *Y *and the covariate *X*_*k*_. If the partial correlation between *X*_*k *_and *Y *vanishes, so will also the two corresponding regression coefficients βky
 MathType@MTEF@5@5@+=feaafiart1ev1aaatCvAUfKttLearuWrP9MDH5MBPbIqV92AaeXatLxBI9gBaebbnrfifHhDYfgasaacH8akY=wiFfYdH8Gipec8Eeeu0xXdbba9frFj0=OqFfea0dXdd9vqai=hGuQ8kuc9pgc9s8qqaq=dirpe0xb9q8qiLsFr0=vr0=vr0dc8meaabaqaciaacaGaaeqabaqabeGadaaakeaaiiGacqWFYoGydaqhaaWcbaGaem4AaSgabaGaemyEaKhaaaaa@315B@ and βyk
 MathType@MTEF@5@5@+=feaafiart1ev1aaatCvAUfKttLearuWrP9MDH5MBPbIqV92AaeXatLxBI9gBaebbnrfifHhDYfgasaacH8akY=wiFfYdH8Gipec8Eeeu0xXdbba9frFj0=OqFfea0dXdd9vqai=hGuQ8kuc9pgc9s8qqaq=dirpe0xb9q8qiLsFr0=vr0=vr0dc8meaabaqaciaacaGaaeqabaqabeGadaaakeaaiiGacqWFYoGydaqhaaWcbaGaemyEaKhabaGaem4AaSgaaaaa@315B@. In a partial correlation graph an edge is drawn between two nodes *Y *and *X*_*k *_if A
 MathType@MTEF@5@5@+=feaafiart1ev1aaatCvAUfKttLearuWrP9MDH5MBPbIqV92AaeXatLxBI9gBaebbnrfifHhDYfgasaacH8akY=wiFfYdH8Gipec8Eeeu0xXdbba9frFj0=OqFfea0dXdd9vqai=hGuQ8kuc9pgc9s8qqaq=dirpe0xb9q8qiLsFr0=vr0=vr0dc8meaabaqaciaacaGaaeqabaqabeGadaaakeaat0uy0HwzTfgDPnwy1egaryqtHrhAL1wy0L2yHvdaiqaaliab=bq8bbaa@382B@ ≠ 0.

ℬ
 MathType@MTEF@5@5@+=feaafiart1ev1aaatCvAUfKttLearuWrP9MDH5MBPbIqV92AaeXatLxBI9gBaebbnrfifHhDYfgasaacH8akY=wiFfYdH8Gipec8Eeeu0xXdbba9frFj0=OqFfea0dXdd9vqai=hGuQ8kuc9pgc9s8qqaq=dirpe0xb9q8qiLsFr0=vr0=vr0dc8meaabaqaciaacaGaaeqabaqabeGadaaakeaat0uy0HwzTfgDPnwy1egaryqtHrhAL1wy0L2yHvdaiqaaliab=Xsicbaa@3788@ : This factor adjusts the regression coefficient for the relative reduction in variance of *Y *and *X*_*k *_due to the respective other covariates. In the algorithm outlined below a test of log(ℬ
 MathType@MTEF@5@5@+=feaafiart1ev1aaatCvAUfKttLearuWrP9MDH5MBPbIqV92AaeXatLxBI9gBaebbnrfifHhDYfgasaacH8akY=wiFfYdH8Gipec8Eeeu0xXdbba9frFj0=OqFfea0dXdd9vqai=hGuQ8kuc9pgc9s8qqaq=dirpe0xb9q8qiLsFr0=vr0=vr0dc8meaabaqaciaacaGaaeqabaqabeGadaaakeaat0uy0HwzTfgDPnwy1egaryqtHrhAL1wy0L2yHvdaiqaaliab=Xsicbaa@3788@) establishes the directionality of edges of a partially causal network.

C
 MathType@MTEF@5@5@+=feaafiart1ev1aaatCvAUfKttLearuWrP9MDH5MBPbIqV92AaeXatLxBI9gBaebbnrfifHhDYfgasaacH8akY=wiFfYdH8Gipec8Eeeu0xXdbba9frFj0=OqFfea0dXdd9vqai=hGuQ8kuc9pgc9s8qqaq=dirpe0xb9q8qiLsFr0=vr0=vr0dc8meaabaqaciaacaGaaeqabaqabeGadaaakeaat0uy0HwzTfgDPnwy1egaryqtHrhAL1wy0L2yHvdaiqaaliab=jq8dbaa@382F@ : This is a scale factor correcting for different units in *Y *and *X*_*k*_.

The product Aℬ=βkyσk2/σy2
 MathType@MTEF@5@5@+=feaafiart1ev1aaatCvAUfKttLearuWrP9MDH5MBPbIqV92AaeXatLxBI9gBaebbnrfifHhDYfgasaacH8akY=wiFfYdH8Gipec8Eeeu0xXdbba9frFj0=OqFfea0dXdd9vqai=hGuQ8kuc9pgc9s8qqaq=dirpe0xb9q8qiLsFr0=vr0=vr0dc8meaabaqaciaacaGaaeqabaqabeGadaaakeaat0uy0HwzTfgDPnwy1egaryqtHrhAL1wy0L2yHvdaiqaacqWFaeFqcqWFSeIqcqGH9aqpiiGacqGFYoGydaqhaaWcbaGaem4AaSgabaGaemyEaKhaaOWaaOaaaeaacqGFdpWCdaqhaaWcbaGaem4AaSgabaGaeGOmaidaaOGaei4la8Iae43Wdm3aa0baaSqaaiabdMha5bqaaiabikdaYaaaaeqaaaaa@4884@ is also known as the standardized regression coefficient. Note that for computing both A
 MathType@MTEF@5@5@+=feaafiart1ev1aaatCvAUfKttLearuWrP9MDH5MBPbIqV92AaeXatLxBI9gBaebbnrfifHhDYfgasaacH8akY=wiFfYdH8Gipec8Eeeu0xXdbba9frFj0=OqFfea0dXdd9vqai=hGuQ8kuc9pgc9s8qqaq=dirpe0xb9q8qiLsFr0=vr0=vr0dc8meaabaqaciaacaGaaeqabaqabeGadaaakeaat0uy0HwzTfgDPnwy1egaryqtHrhAL1wy0L2yHvdaiqaaliab=bq8bbaa@382B@ and ℬ
 MathType@MTEF@5@5@+=feaafiart1ev1aaatCvAUfKttLearuWrP9MDH5MBPbIqV92AaeXatLxBI9gBaebbnrfifHhDYfgasaacH8akY=wiFfYdH8Gipec8Eeeu0xXdbba9frFj0=OqFfea0dXdd9vqai=hGuQ8kuc9pgc9s8qqaq=dirpe0xb9q8qiLsFr0=vr0=vr0dc8meaabaqaciaacaGaaeqabaqabeGadaaakeaat0uy0HwzTfgDPnwy1egaryqtHrhAL1wy0L2yHvdaiqaaliab=Xsicbaa@3788@ only the correlation matrix is needed, as the variance information is already accounted for by the third factor C
 MathType@MTEF@5@5@+=feaafiart1ev1aaatCvAUfKttLearuWrP9MDH5MBPbIqV92AaeXatLxBI9gBaebbnrfifHhDYfgasaacH8akY=wiFfYdH8Gipec8Eeeu0xXdbba9frFj0=OqFfea0dXdd9vqai=hGuQ8kuc9pgc9s8qqaq=dirpe0xb9q8qiLsFr0=vr0=vr0dc8meaabaqaciaacaGaaeqabaqabeGadaaakeaat0uy0HwzTfgDPnwy1egaryqtHrhAL1wy0L2yHvdaiqaaliab=jq8dbaa@382F@.

In this context it is also helpful to recall the diverse statistical interpretations of SPV:

• SPV is the *proportion *of variance that remains (unexplained) after regressing against all other variables.

• For the OLS estimator SPV is equal to 1 - *R*^2^, where *R *is the usual coefficient of determination.

• SPV is the inverse of the diagonal of the inverse of the *correlation *matrix. Thus, if there is no correlation (unit diagonal correlation matrix) the partial variance equals the variance, and hence SPV = 1.

• SPV may also be estimated by 1/VIF, where VIF is the usual variance inflation factor [cf. [26]].

### Heuristic algorithm for discovering approximate causal networks

The above decomposition (Equation 2) suggests the following simple strategy for statistical learning of causal networks. First, by multiple testing of A
 MathType@MTEF@5@5@+=feaafiart1ev1aaatCvAUfKttLearuWrP9MDH5MBPbIqV92AaeXatLxBI9gBaebbnrfifHhDYfgasaacH8akY=wiFfYdH8Gipec8Eeeu0xXdbba9frFj0=OqFfea0dXdd9vqai=hGuQ8kuc9pgc9s8qqaq=dirpe0xb9q8qiLsFr0=vr0=vr0dc8meaabaqaciaacaGaaeqabaqabeGadaaakeaat0uy0HwzTfgDPnwy1egaryqtHrhAL1wy0L2yHvdaiqaaliab=bq8bbaa@382B@ = 0 we determine the network topology, i.e. we identify those edges for which the corresponding partial correlation is not vanishing. Second, by subsequent multiple testing of log(ℬ
 MathType@MTEF@5@5@+=feaafiart1ev1aaatCvAUfKttLearuWrP9MDH5MBPbIqV92AaeXatLxBI9gBaebbnrfifHhDYfgasaacH8akY=wiFfYdH8Gipec8Eeeu0xXdbba9frFj0=OqFfea0dXdd9vqai=hGuQ8kuc9pgc9s8qqaq=dirpe0xb9q8qiLsFr0=vr0=vr0dc8meaabaqaciaacaGaaeqabaqabeGadaaakeaat0uy0HwzTfgDPnwy1egaryqtHrhAL1wy0L2yHvdaiqaaliab=Xsicbaa@3788@) = 0 we establish a partial ordering of the nodes, which in turn imposes a partial directionality upon the edges.

In more detail, we propose the following five-step algorithm:

1. First, it is essential to determine an accurate and positive definite estimate ***R ***of the correlation matrix. Only if the sample size is large with many more observations than variables (*n *> > *p*) the usual empirical correlation estimate will be suitable. In all other instances, the use of a regularized estimator is absolutely vital (e.g., the Stein-type shrinkage estimator of [20]) in order to improve efficiency and to guarantee positive definiteness. In addition, if the samples are longitudinal it may be necessary to adjust for autocorrelation [27].

2. From the estimated correlations we compute the partial variances and correlations (see Table 1), and from those in turn plug-in estimates of the factors A
 MathType@MTEF@5@5@+=feaafiart1ev1aaatCvAUfKttLearuWrP9MDH5MBPbIqV92AaeXatLxBI9gBaebbnrfifHhDYfgasaacH8akY=wiFfYdH8Gipec8Eeeu0xXdbba9frFj0=OqFfea0dXdd9vqai=hGuQ8kuc9pgc9s8qqaq=dirpe0xb9q8qiLsFr0=vr0=vr0dc8meaabaqaciaacaGaaeqabaqabeGadaaakeaat0uy0HwzTfgDPnwy1egaryqtHrhAL1wy0L2yHvdaiqaaliab=bq8bbaa@382B@ and ℬ
 MathType@MTEF@5@5@+=feaafiart1ev1aaatCvAUfKttLearuWrP9MDH5MBPbIqV92AaeXatLxBI9gBaebbnrfifHhDYfgasaacH8akY=wiFfYdH8Gipec8Eeeu0xXdbba9frFj0=OqFfea0dXdd9vqai=hGuQ8kuc9pgc9s8qqaq=dirpe0xb9q8qiLsFr0=vr0=vr0dc8meaabaqaciaacaGaaeqabaqabeGadaaakeaat0uy0HwzTfgDPnwy1egaryqtHrhAL1wy0L2yHvdaiqaaliab=Xsicbaa@3788@ of Equation 2 for all possible edges. Note that in this calculation each variable assumes in turn the role of the response *Y *. An efficient way to calculate the various ℬ
 MathType@MTEF@5@5@+=feaafiart1ev1aaatCvAUfKttLearuWrP9MDH5MBPbIqV92AaeXatLxBI9gBaebbnrfifHhDYfgasaacH8akY=wiFfYdH8Gipec8Eeeu0xXdbba9frFj0=OqFfea0dXdd9vqai=hGuQ8kuc9pgc9s8qqaq=dirpe0xb9q8qiLsFr0=vr0=vr0dc8meaabaqaciaacaGaaeqabaqabeGadaaakeaat0uy0HwzTfgDPnwy1egaryqtHrhAL1wy0L2yHvdaiqaaliab=Xsicbaa@3788@ is given by taking the square root of the diagonal of the inverse of the estimated correlation matrix, and computing the corresponding pairwise ratios.

3. Subsequently, we infer the partial correlation graph following the algorithm described in [19]. Essentially, we perform multiple testing of all partial correlation coefficients A
 MathType@MTEF@5@5@+=feaafiart1ev1aaatCvAUfKttLearuWrP9MDH5MBPbIqV92AaeXatLxBI9gBaebbnrfifHhDYfgasaacH8akY=wiFfYdH8Gipec8Eeeu0xXdbba9frFj0=OqFfea0dXdd9vqai=hGuQ8kuc9pgc9s8qqaq=dirpe0xb9q8qiLsFr0=vr0=vr0dc8meaabaqaciaacaGaaeqabaqabeGadaaakeaat0uy0HwzTfgDPnwy1egaryqtHrhAL1wy0L2yHvdaiqaaliab=bq8bbaa@382B@. Note that for high dimensions (large *p*) the null distribution of partial correlations across edges can be determined from the data, which in turn allows the adaptive computation of corresponding false discovery rates [28].

4. In a similar fashion we then conduct multiple testing of all log(ℬ
 MathType@MTEF@5@5@+=feaafiart1ev1aaatCvAUfKttLearuWrP9MDH5MBPbIqV92AaeXatLxBI9gBaebbnrfifHhDYfgasaacH8akY=wiFfYdH8Gipec8Eeeu0xXdbba9frFj0=OqFfea0dXdd9vqai=hGuQ8kuc9pgc9s8qqaq=dirpe0xb9q8qiLsFr0=vr0=vr0dc8meaabaqaciaacaGaaeqabaqabeGadaaakeaat0uy0HwzTfgDPnwy1egaryqtHrhAL1wy0L2yHvdaiqaaliab=Xsicbaa@3788@). As ℬ
 MathType@MTEF@5@5@+=feaafiart1ev1aaatCvAUfKttLearuWrP9MDH5MBPbIqV92AaeXatLxBI9gBaebbnrfifHhDYfgasaacH8akY=wiFfYdH8Gipec8Eeeu0xXdbba9frFj0=OqFfea0dXdd9vqai=hGuQ8kuc9pgc9s8qqaq=dirpe0xb9q8qiLsFr0=vr0=vr0dc8meaabaqaciaacaGaaeqabaqabeGadaaakeaat0uy0HwzTfgDPnwy1egaryqtHrhAL1wy0L2yHvdaiqaaliab=Xsicbaa@3788@ is the ratio of two variances with the same degrees of freedom, it is implicit that log(ℬ
 MathType@MTEF@5@5@+=feaafiart1ev1aaatCvAUfKttLearuWrP9MDH5MBPbIqV92AaeXatLxBI9gBaebbnrfifHhDYfgasaacH8akY=wiFfYdH8Gipec8Eeeu0xXdbba9frFj0=OqFfea0dXdd9vqai=hGuQ8kuc9pgc9s8qqaq=dirpe0xb9q8qiLsFr0=vr0=vr0dc8meaabaqaciaacaGaaeqabaqabeGadaaakeaat0uy0HwzTfgDPnwy1egaryqtHrhAL1wy0L2yHvdaiqaaliab=Xsicbaa@3788@) is approximately normally distributed [29], with an unknown variance parameter *θ*. Thus, the observed *z *= log(ℬ
 MathType@MTEF@5@5@+=feaafiart1ev1aaatCvAUfKttLearuWrP9MDH5MBPbIqV92AaeXatLxBI9gBaebbnrfifHhDYfgasaacH8akY=wiFfYdH8Gipec8Eeeu0xXdbba9frFj0=OqFfea0dXdd9vqai=hGuQ8kuc9pgc9s8qqaq=dirpe0xb9q8qiLsFr0=vr0=vr0dc8meaabaqaciaacaGaaeqabaqabeGadaaakeaat0uy0HwzTfgDPnwy1egaryqtHrhAL1wy0L2yHvdaiqaaliab=Xsicbaa@3788@) across all edges follow a mixture distribution

*f*(*z*) = *η*_0 _*N*(0, *θ*) + (1 - *η*_0_) *f*_*A *_(*z*).

Assuming that most *z *belong to the null model, i.e. that most edges are undirected, it is possible to infer non-parametrically the alternative distribution *f*_*A *_(*z*), the proportion *η*_0_, as well as the variance parameter *θ *– for an algorithm see [28]. From the resulting densities and distribution functions local and tail-area-based false discovery rates for the test log(ℬ
 MathType@MTEF@5@5@+=feaafiart1ev1aaatCvAUfKttLearuWrP9MDH5MBPbIqV92AaeXatLxBI9gBaebbnrfifHhDYfgasaacH8akY=wiFfYdH8Gipec8Eeeu0xXdbba9frFj0=OqFfea0dXdd9vqai=hGuQ8kuc9pgc9s8qqaq=dirpe0xb9q8qiLsFr0=vr0=vr0dc8meaabaqaciaacaGaaeqabaqabeGadaaakeaat0uy0HwzTfgDPnwy1egaryqtHrhAL1wy0L2yHvdaiqaaliab=Xsicbaa@3788@) = 0 are computed. Note that in this procedure we include all edges, regardless of the corresponding value of A
 MathType@MTEF@5@5@+=feaafiart1ev1aaatCvAUfKttLearuWrP9MDH5MBPbIqV92AaeXatLxBI9gBaebbnrfifHhDYfgasaacH8akY=wiFfYdH8Gipec8Eeeu0xXdbba9frFj0=OqFfea0dXdd9vqai=hGuQ8kuc9pgc9s8qqaq=dirpe0xb9q8qiLsFr0=vr0=vr0dc8meaabaqaciaacaGaaeqabaqabeGadaaakeaat0uy0HwzTfgDPnwy1egaryqtHrhAL1wy0L2yHvdaiqaaliab=bq8bbaa@382B@ or the outcome of the test A
 MathType@MTEF@5@5@+=feaafiart1ev1aaatCvAUfKttLearuWrP9MDH5MBPbIqV92AaeXatLxBI9gBaebbnrfifHhDYfgasaacH8akY=wiFfYdH8Gipec8Eeeu0xXdbba9frFj0=OqFfea0dXdd9vqai=hGuQ8kuc9pgc9s8qqaq=dirpe0xb9q8qiLsFr0=vr0=vr0dc8meaabaqaciaacaGaaeqabaqabeGadaaakeaat0uy0HwzTfgDPnwy1egaryqtHrhAL1wy0L2yHvdaiqaaliab=bq8bbaa@382B@ = 0.

5. Finally, a partially directed network is constructed as follows. All edges in the correlation graph with significant log(ℬ
 MathType@MTEF@5@5@+=feaafiart1ev1aaatCvAUfKttLearuWrP9MDH5MBPbIqV92AaeXatLxBI9gBaebbnrfifHhDYfgasaacH8akY=wiFfYdH8Gipec8Eeeu0xXdbba9frFj0=OqFfea0dXdd9vqai=hGuQ8kuc9pgc9s8qqaq=dirpe0xb9q8qiLsFr0=vr0=vr0dc8meaabaqaciaacaGaaeqabaqabeGadaaakeaat0uy0HwzTfgDPnwy1egaryqtHrhAL1wy0L2yHvdaiqaaliab=Xsicbaa@3788@) ≠ 0 are directed in such a fashion that the direction of the arrow points from the node with the larger standardized partial variance (the more "exogenous" variable) to the node with the smaller standardized partial variance (the more "endogenous" variable). The other edges with log(ℬ
 MathType@MTEF@5@5@+=feaafiart1ev1aaatCvAUfKttLearuWrP9MDH5MBPbIqV92AaeXatLxBI9gBaebbnrfifHhDYfgasaacH8akY=wiFfYdH8Gipec8Eeeu0xXdbba9frFj0=OqFfea0dXdd9vqai=hGuQ8kuc9pgc9s8qqaq=dirpe0xb9q8qiLsFr0=vr0=vr0dc8meaabaqaciaacaGaaeqabaqabeGadaaakeaat0uy0HwzTfgDPnwy1egaryqtHrhAL1wy0L2yHvdaiqaaliab=Xsicbaa@3788@) ≈ 0 remain undirected. The subgraph consisting of all directed edges constitutes the inferred causal network. Note that this does not necessarily include all nodes that are contained in the GGM network.

## Results and discussion

### Interpretation of the resulting graph

The above algorithm returns a partially directed partial correlation graph, whose directed edges form a causal network.

This procedure can be motivated by the following connection between partial correlation graph and a system of linear equations, where each node is in turn taken as a response variable and regressed against all other remaining nodes. In this setting the partial correlation coefficient is the geometric mean of βky
 MathType@MTEF@5@5@+=feaafiart1ev1aaatCvAUfKttLearuWrP9MDH5MBPbIqV92AaeXatLxBI9gBaebbnrfifHhDYfgasaacH8akY=wiFfYdH8Gipec8Eeeu0xXdbba9frFj0=OqFfea0dXdd9vqai=hGuQ8kuc9pgc9s8qqaq=dirpe0xb9q8qiLsFr0=vr0=vr0dc8meaabaqaciaacaGaaeqabaqabeGadaaakeaaiiGacqWFYoGydaqhaaWcbaGaem4AaSgabaGaemyEaKhaaaaa@315B@ and the corresponding reciprocal coefficient βyk
 MathType@MTEF@5@5@+=feaafiart1ev1aaatCvAUfKttLearuWrP9MDH5MBPbIqV92AaeXatLxBI9gBaebbnrfifHhDYfgasaacH8akY=wiFfYdH8Gipec8Eeeu0xXdbba9frFj0=OqFfea0dXdd9vqai=hGuQ8kuc9pgc9s8qqaq=dirpe0xb9q8qiLsFr0=vr0=vr0dc8meaabaqaciaacaGaaeqabaqabeGadaaakeaaiiGacqWFYoGydaqhaaWcbaGaemyEaKhabaGaem4AaSgaaaaa@315B@, i.e.

βykβky=|ρ˜yk|
 MathType@MTEF@5@5@+=feaafiart1ev1aaatCvAUfKttLearuWrP9MDH5MBPbIqV92AaeXatLxBI9gBaebbnrfifHhDYfgasaacH8akY=wiFfYdH8Gipec8Eeeu0xXdbba9frFj0=OqFfea0dXdd9vqai=hGuQ8kuc9pgc9s8qqaq=dirpe0xb9q8qiLsFr0=vr0=vr0dc8meaabaqaciaacaGaaeqabaqabeGadaaakeaadaGcaaqaaGGaciab=j7aInaaDaaaleaacqWG5bqEaeaacqWGRbWAaaGccqWFYoGydaqhaaWcbaGaem4AaSgabaGaemyEaKhaaaqabaGccqGH9aqpdaabdaqaaiqb=f8aYzaaiaWaaSbaaSqaaiabdMha5jabdUgaRbqabaaakiaawEa7caGLiWoaaaa@3F24@

[see also equation 16 of ref. [20]]. In this light, an undirected edge between two nodes A and B in a partial correlation graph may also be interpreted as bidirected edge, in the sense that A influences B and vice versa in the underlying system of regression. Therefore, the test ℬ
 MathType@MTEF@5@5@+=feaafiart1ev1aaatCvAUfKttLearuWrP9MDH5MBPbIqV92AaeXatLxBI9gBaebbnrfifHhDYfgasaacH8akY=wiFfYdH8Gipec8Eeeu0xXdbba9frFj0=OqFfea0dXdd9vqai=hGuQ8kuc9pgc9s8qqaq=dirpe0xb9q8qiLsFr0=vr0=vr0dc8meaabaqaciaacaGaaeqabaqabeGadaaakeaat0uy0HwzTfgDPnwy1egaryqtHrhAL1wy0L2yHvdaiqaaliab=Xsicbaa@3788@ = 1 can be understood as *removing *one of these two directions, where Equation 2 suggests that only the relative variance reduction between the two involved nodes needs to be considered for establishing the final direction.

### Reconstruction efficiency and approximations underlying the algorithm

#### Topology of the network

The proposed algorithm is an extension of the GGM inference approach of [19,20]. Its accuracy of correctly recovering the *topology *of the partial correlation graph has been established, e.g., in [30]. 

However, it is well known that a directed Bayesian network and the corresponding undirected graph are not necessarily topologically identical: in the undirected graph for computing the partial correlations one conditions on all other nodes whereas in the directed graph one conditions only on a subset of nodes, in order to avoid conditioning "on the future" (i.e. on the dependent nodes). Therefore, it is critical to evaluate to what extent full order partial correlations are reasonable approximations for lower order partial correlations. This has already been investigated intensively by [31] who showed that in certain situations (sparse graphs, faithfulness assumption etc.) lower order partial correlations may be used as approximate substitute of full conditional correlations. Therefore, in the proposed algorithm we adopt the very same argument but apply it in the different direction, i.e. we approximate lower order partial correlation by full order partial correlation.

#### Node ordering

A second approximation implicit in our algorithm concerns the determination of the ordering of the nodes, which is done by multiple testing of pairwise ratios of standardized partial variances. We have conducted a number of numerical simulations (data not shown) that indicate that for randomly simulated DAGs the ordering of the nodes is indeed well reflected in the partial variances, as expected.

However, from variable selection in linear models it is also known that the partial variance (or the related *R*^2^) may not always be a reliable indicator for variable importance. Nevertheless, the partial ordering of nodes according to SPV and the implicit model selection in the underlying regressions is a very different procedure in comparison to the standard variable selection approaches, in which the increase or decrease of the *R*^2 ^is taken as indicator of whether or not a variable is to be included, or a decomposition of *R*^2 ^is sought [for a review see, e.g., [32]]. The distinctive feature of our procedure is that by performing all tests log(ℬ
 MathType@MTEF@5@5@+=feaafiart1ev1aaatCvAUfKttLearuWrP9MDH5MBPbIqV92AaeXatLxBI9gBaebbnrfifHhDYfgasaacH8akY=wiFfYdH8Gipec8Eeeu0xXdbba9frFj0=OqFfea0dXdd9vqai=hGuQ8kuc9pgc9s8qqaq=dirpe0xb9q8qiLsFr0=vr0=vr0dc8meaabaqaciaacaGaaeqabaqabeGadaaakeaat0uy0HwzTfgDPnwy1egaryqtHrhAL1wy0L2yHvdaiqaaliab=Xsicbaa@3788@) ≠ 0 simultaneously we consider all *p *regression equations at once, even if the final feature selection occurs only locally on the level of an individual regression.

It is also noteworthy that, as we impose directionality from the less well explained variable (large SPV, "exogenous", "independent") to the one with relatively lower SPV (well explained, "endogenous", "dependent" variable), we effectively choose the direction with the relatively *smaller *regression coefficient (conditional that the corresponding partial correlation is also significant).

### Further properties of the heuristic algorithm and of the resulting graphs

The simple heuristic network discovery algorithm exhibits a number of further properties worth noting:

1. The estimated partially directed network cannot contain any (partially) directed cycles. For instance, it is not possible for a graph to contain a pattern such as *A *→ *B *→ *A*. This example would imply SPV_*A *_> SPV_*B *_> SPV_*A*_, which is a contradiction. As a consequence, the subgraph containing the directed edges only is also acyclic (and hence a DAG).

2. The assignment of directionality is transitive. If there is a directed edge from *A *to *B *and from *B *to *C *then there must also be a directed edge from *A *to *C*. Note however, that actual inclusion of a directed edge into the causal network is conditional on a non-zero partial correlation coefficient.

3. As the algorithm relies on correlations as input, causal processes that produce the same correlation matrix lead to the same inferred graph, and hence are indistinguishable. The existence of such equivalence classes is well known for SEMs [33] and also for Bayesian belief networks [34].

4. The proposed algorithm is scale-invariant by construction. Hence, a (linear) change in any of units of the data has no effect on the overall estimated partially directed network, and the implied causal relations.

5. We emphasize that the partially directed network is *not *the chain graph representing the equivalence class of the causal network that is obtained by considering only its directed edges – see [34].

6. The computational complexity of the algorithm is *O*(*p*^3^). Hence, it is no more expensive than computing the partial correlation graph, and thus allows for estimation of networks containing in the order of thousands and more nodes.

### Analysis of a plant expression data set

To illustrate our algorithm for discovering causal structure, we applied the approach to a real world data example. Specifically, we reanalyzed expression time series resulting from an experiment investigating the impact of the diurnal cycle on the starch metabolism of *Arabidopsis thaliana *[35]. This is the same data set we used in a sister paper concerning the estimation of a vector autoregressive model [36].

The data are gene expression time series measurements collected at 11 different time points (0, 1, 2, 4, 8, 12, 13, 14, 16, 20, and 24 hours after the start of the experiment). The corresponding calibrated signal intensities for 22,814 genes/probe sets and for two biological replicates are available from the NASCArrays repository, experiment no. 60 [37]. After log-transforming the data we filtered out all genes containing missing values and whose maximum signal intensity value was lower than 5 on a log-base 2 scale. Subsequently, we applied the periodicity test of [38] to identify the probes associated with the day-night cycle. As a result, a subset of 800 genes remained for further analysis.

**Figure 1:**
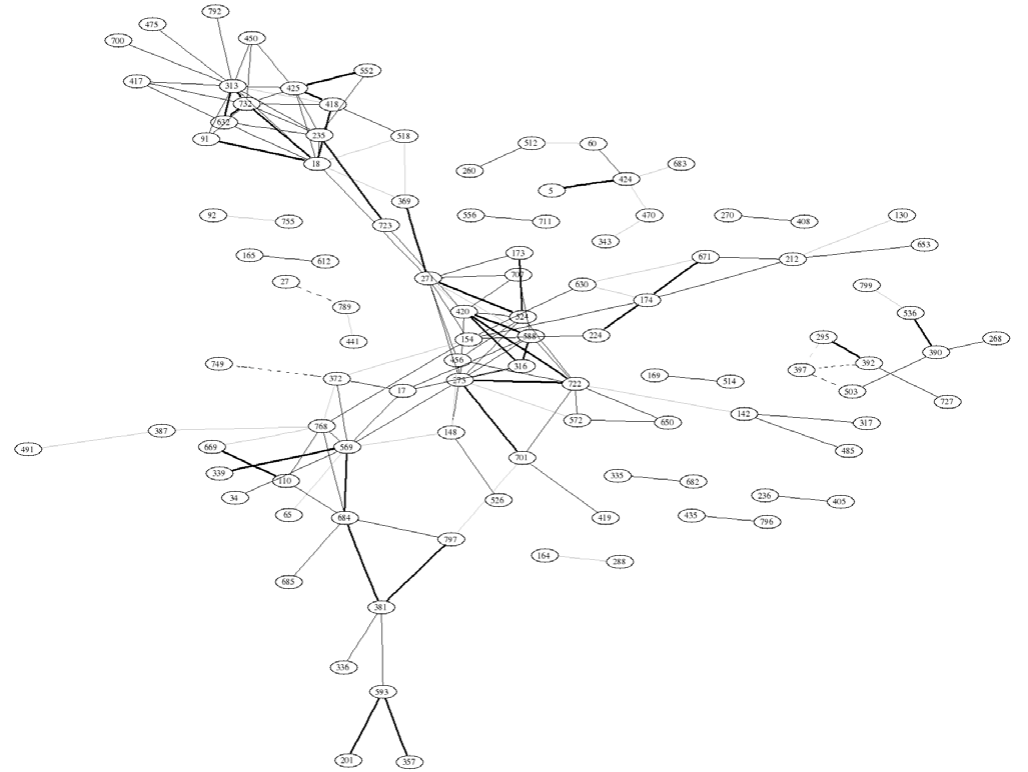
Correlation network inferred from the *Arabidopsis thaliana *data. The solid and dotted lines indicate positive and negative correlation coefficients, respectively, and the line intensity denotes their strength. The network displays the 150 edges with the largest absolute correlation. For annotation of the nodes in this graph see the electronic information contained in the R package "GeneNet" [40] and the original data paper [35].

In order to estimate the correlation matrix for the 800 genes described by the data set we employed the dynamical correlation shrinkage estimator of [39] as this takes account of the autocorrelation. The corresponding correlation graph is displayed in Figure 1. It shows the 150 edges with the largest absolute values of correlation. This graph is very hard to interpret, the branches do not have any immediate or intuitive meaning (a complete annotation of the nodes can be found along with the dataset itself in the R package "GeneNet" [40]). For instance, there are no hubs as typically observed in biological networks [41,42].

**Figure 2:**
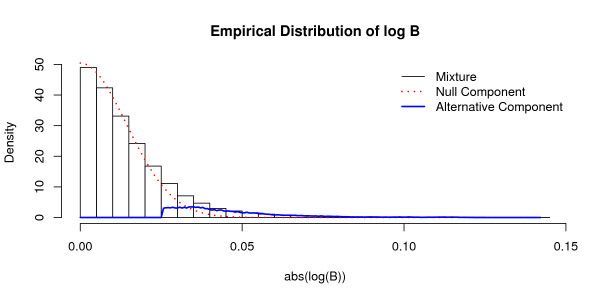
Distribution of log ℬ
 MathType@MTEF@5@5@+=feaafiart1ev1aaatCvAUfKttLearuWrP9MDH5MBPbIqV92AaeXatLxBI9gBaebbnrfifHhDYfgasaacH8akY=wiFfYdH8Gipec8Eeeu0xXdbba9frFj0=OqFfea0dXdd9vqai=hGuQ8kuc9pgc9s8qqaq=dirpe0xb9q8qiLsFr0=vr0=vr0dc8meaabaqaciaacaGaaeqabaqabeGadaaakeaat0uy0HwzTfgDPnwy1egaryqtHrhAL1wy0L2yHvdaiqaaliab=Xsicbaa@3788@ for the *Arabidopsis thaliana *data. The null distribution is depicted by the dashed line; it follows a normal distribution with zero mean and a standard deviation of 0.014. The solid line signifies the alternative distribution. The empirical distribution (indicated by the histogram) is composed of the null distribution (*η*_0 _= 0.8995) and of the alternative distribution (*η*_*A *_= 0.1005).

This is in great contrast to the partially directed partial correlation graph. For this specific data set, by multiple testing of the factor A
 MathType@MTEF@5@5@+=feaafiart1ev1aaatCvAUfKttLearuWrP9MDH5MBPbIqV92AaeXatLxBI9gBaebbnrfifHhDYfgasaacH8akY=wiFfYdH8Gipec8Eeeu0xXdbba9frFj0=OqFfea0dXdd9vqai=hGuQ8kuc9pgc9s8qqaq=dirpe0xb9q8qiLsFr0=vr0=vr0dc8meaabaqaciaacaGaaeqabaqabeGadaaakeaat0uy0HwzTfgDPnwy1egaryqtHrhAL1wy0L2yHvdaiqaaliab=bq8bbaa@382B@ we identified 6, 102 significant edges connecting 669 nodes. For the second factor ℬ
 MathType@MTEF@5@5@+=feaafiart1ev1aaatCvAUfKttLearuWrP9MDH5MBPbIqV92AaeXatLxBI9gBaebbnrfifHhDYfgasaacH8akY=wiFfYdH8Gipec8Eeeu0xXdbba9frFj0=OqFfea0dXdd9vqai=hGuQ8kuc9pgc9s8qqaq=dirpe0xb9q8qiLsFr0=vr0=vr0dc8meaabaqaciaacaGaaeqabaqabeGadaaakeaat0uy0HwzTfgDPnwy1egaryqtHrhAL1wy0L2yHvdaiqaaliab=Xsicbaa@3788@, determined whether edges are directed, the distribution of log(ℬ
 MathType@MTEF@5@5@+=feaafiart1ev1aaatCvAUfKttLearuWrP9MDH5MBPbIqV92AaeXatLxBI9gBaebbnrfifHhDYfgasaacH8akY=wiFfYdH8Gipec8Eeeu0xXdbba9frFj0=OqFfea0dXdd9vqai=hGuQ8kuc9pgc9s8qqaq=dirpe0xb9q8qiLsFr0=vr0=vr0dc8meaabaqaciaacaGaaeqabaqabeGadaaakeaat0uy0HwzTfgDPnwy1egaryqtHrhAL1wy0L2yHvdaiqaaliab=Xsicbaa@3788@) is displayed in Figure 2. The null distribution (dashed line) follows a normal distribution and characterizes the edges that cannot be directed. The alternative distribution (solid line) coincides with the directed edges. In total, we found 15, 928 significant directions.

To construct the network, we projected upon the significant edges (factor A
 MathType@MTEF@5@5@+=feaafiart1ev1aaatCvAUfKttLearuWrP9MDH5MBPbIqV92AaeXatLxBI9gBaebbnrfifHhDYfgasaacH8akY=wiFfYdH8Gipec8Eeeu0xXdbba9frFj0=OqFfea0dXdd9vqai=hGuQ8kuc9pgc9s8qqaq=dirpe0xb9q8qiLsFr0=vr0=vr0dc8meaabaqaciaacaGaaeqabaqabeGadaaakeaat0uy0HwzTfgDPnwy1egaryqtHrhAL1wy0L2yHvdaiqaaliab=bq8bbaa@382B@) the significant directions (factor ℬ
 MathType@MTEF@5@5@+=feaafiart1ev1aaatCvAUfKttLearuWrP9MDH5MBPbIqV92AaeXatLxBI9gBaebbnrfifHhDYfgasaacH8akY=wiFfYdH8Gipec8Eeeu0xXdbba9frFj0=OqFfea0dXdd9vqai=hGuQ8kuc9pgc9s8qqaq=dirpe0xb9q8qiLsFr0=vr0=vr0dc8meaabaqaciaacaGaaeqabaqabeGadaaakeaat0uy0HwzTfgDPnwy1egaryqtHrhAL1wy0L2yHvdaiqaaliab=Xsicbaa@3788@). In the network of significant associations, 1,216 directions were significant. Note that the fraction of significant directions is by far greater in the subset of the significant partial correlations than in the complete set of all partial correlations. This agrees with the intuitive notion, that causal influences can only be attributed to existing connections between variables.

**Figure 3:**
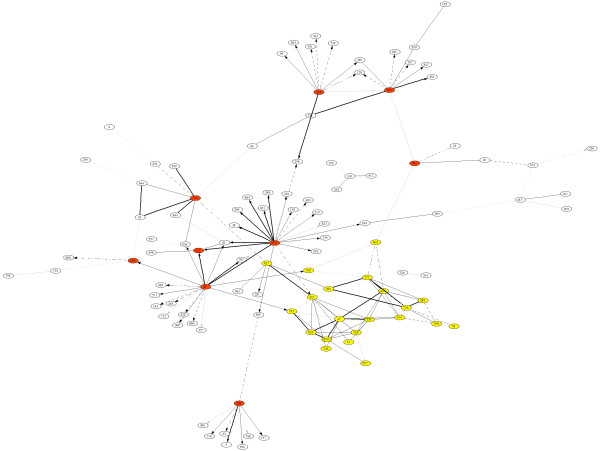
Partially causal network inferred from the *Arabidopsis thaliana *data by the method introduced in this paper – note the difference to the correlation network of Figure 1. The topology of the partially causal network is identical to that of a partial correlation graph (GGM, CIG). However, edges with significant directionality (as indicated by a factor ℬ
 MathType@MTEF@5@5@+=feaafiart1ev1aaatCvAUfKttLearuWrP9MDH5MBPbIqV92AaeXatLxBI9gBaebbnrfifHhDYfgasaacH8akY=wiFfYdH8Gipec8Eeeu0xXdbba9frFj0=OqFfea0dXdd9vqai=hGuQ8kuc9pgc9s8qqaq=dirpe0xb9q8qiLsFr0=vr0=vr0dc8meaabaqaciaacaGaaeqabaqabeGadaaakeaat0uy0HwzTfgDPnwy1egaryqtHrhAL1wy0L2yHvdaiqaaliab=Xsicbaa@3788@ that is significantly smaller or larger than one) are oriented.

The resulting partially causal network is shown in Figure 3. For reasons of clarity we show only the subnetwork containing the 150 most significant edges, which connect 107 nodes. This graph exhibits a clear "hub" connectivity structure (nodes filled with red color). A prominent example for this is node 570, others are 81, 558, 783 and a few more genes. We see that many of the hub nodes have mostly outgoing arcs, which is indicative for key regulatory genes. This applies, e.g., to node 570, an AP2 transcription factor, or to node 81, a gene involved in DNA-directed RNA polymerase. An interesting aspect of the partially causal network is the web of highly connected genes (colored yellow in the lower right corner of Figure 3), which we hypothesize to constitute some form of a functional module. In this module, it is not possible to determine any directions, which could be due to complex interactions among the nodes of the module. Node 627 is another hub in the network that connects the functional module with the rest of the network and which according to the annotation of [35] encodes a protein of unknown function.

We also see that the partially directed network contains both directed and undirected nodes. This is a distinct advantage of the present approach. Unlike, e.g., a vector autoregressive model [36], it does not *force *directions onto the edges.

Finally, in order to investigate the stability of the inferred partial causal network, we randomly removed data points from the sample, and repeatedly reconstructed the network from the reduced data set. In all cases the general topological structure of the network remained intact, which indicates that this is a signal inherent in the data. This is also confirmed by the analysis using vector autoregressions [36].

## Conclusion

Methods for exploring causal structures in high-dimensional data are growing in importance, particularly in the study of complex biological, medical and financial systems. As a first (and often only) analysis step these data are explored using correlation networks.

Here we have suggested a simple heuristic algorithm that, starting from a (positive definite) correlation matrix, infers a partially directed network that in turn allows generating causal hypotheses of how the data were generated. Our approach is approximate, but it allows analysis of high-dimensional small sampled data, and its computational complexity is very modest. Thus, our heuristic is likely to be applicable whenever a correlation network is computed, and therefore is suitable for screening large-scale data set for causal structure.

Nevertheless, there a several lines along which this method could be extended. For instance, non-linear effects could be accounted for by employing entropy criteria, or by using higher order moments [16]. Furthermore, more sophisticated algorithms may be used to enhance the approximation of lower order partial correlations or the inference of the ordering of the nodes. However, ultimately this would lead to a method similar to the PC algorithm [14,15].

Note that the PC algorithm is more refined than our algorithm, primarily due to additional steps that aim at removing spurious edges (i.e. those edges that are induced between otherwise uncorrelated parent nodes by conditioning on a common child node). However, these iterative refinements may be very time consuming, in particular for high-dimensional graphs.

In contrast, our procedure is non-iterative and therefore both computationally and algorithmically (nearly) as simple as a correlation network. Nevertheless, it still enables the discovery of partially directed processes underlying the data.

In summary, we recommend our approach as a procedure for exploratory screening for causal mechanisms. Subsequently, the resulting hypotheses may then form the basis for more refined analyzes, such as full Bayesian network modeling.

## Authors' contributions

Both authors participated in the development of the methodology and wrote the manuscript. RO carried out all analyzes. All authors approved of the final version of the manuscript.

## Availability and requirements

The method is implemented in the "GeneNet" R package (version 1.2.0), available from CRAN and from . The software includes an R script for reproducing the network analysis of the *Arabidopsis thaliana *data.
